# Computational Insights into the Relationship Between Solution Concentration and Adsorption Energy

**DOI:** 10.3390/molecules31050904

**Published:** 2026-03-09

**Authors:** Wangyan Lv, Wenjie Zhou, Ming Nie, Chenyang Yao, Zhong’ao Wang, Yongchun Liang, Songyu Xie, Chaofang Dong

**Affiliations:** 1 Guangdong Provincial Key Laboratory of Electric Power Equipment Reliability, Electric Power Research Institute of Guangdong Power Grid Co., Ltd., Guangzhou 510080, China; nieming@dky.gd.csg.cn (M.N.); wangzhongao@dky.gd.csg.cn (Z.W.); liangyongchun@dky.gd.csg.cn (Y.L.); xiesongyu@dky.gd.csg.cn (S.X.); 2 Institute for Advanced Materials and Technology, University of Science and Technology Beijing, Beijing 100083, China; asd9998876q@gmail.com (W.Z.); yaochenyang@xs.ustb.edu.cn (C.Y.); cfdong@ustb.edu.cn (C.D.)

**Keywords:** DFT calculation, aggressive ions, adsorption energy, work function, solution concentration

## Abstract

The electrochemical interaction between aggressive ions and metals plays a key role in corrosion failure processes. The Langmuir adsorption isotherm equation was employed to reveal that surface coverage remains largely unchanged at higher concentrations, with the concentration effect partially mediated by the dielectric properties of the solution. The work function and adsorption energy of two typical corrosive elements, Cl and S, adsorbed on the surfaces of two metals (Al and Cu) were systematically calculated. By adjusting solubilization parameters in different implicit solvent models, variations in dielectric properties at similar surface coverage under different concentrations were simulated. It was observed that as the solution concentration increased, the electrostatic shielding effect of the surface solution was enhanced, while the changes in adsorption energy were not statistically significant. However, the work function was found to increase by approximately 20–90 meV with increasing concentration, with the magnitude of this increase dependent on the metal type and surface orientation. This enhancement further strengthened the adsorbate–substrate interaction, thereby influencing the electrochemical reaction kinetics of the surface material.

## 1. Introduction

For most metallic materials, the most critical aspect affecting corrosion is the interaction with aggressive ions [1,2,3]. For passive metals, the greater the interaction with aggressive ions, the easier it is for ions to penetrate into the passivation film [4,5,6], subsequently promoting the breakdown of the passivation film and leading to metal corrosion failure. For active dissolving metals, ion adsorption on the surface can significantly affect properties such as the work function and chemical activity of the surface [3,7,8].

This study aims to delve into the impact of adsorption on the properties of metal surfaces by integrating existing data with previously calculated surface work function data. To fully comprehend the changes in surface properties, a series of computational methods are employed, including adsorption simulations conducted in a vacuum environment and using an implicit solvent model.

In actual solution systems, the relationship between surface coverage and solution concentration is often described through the Langmuir adsorption isotherm curve, and the traditional Langmuir equation [9,10] for calculating concentration and surface coverage is as follows:

(1) $$\theta =\frac{q_{e}}{q_{0}}=\frac{Kc_{e}}{1+Kc_{e}}$$

where *θ* is the coverage at equilibrium, *q_e_* is the amount of adsorbed substance at equilibrium; *K* is the Langmuir adsorption equilibrium constant; *q*_0_ represents the maximum amount of substance that can be adsorbed in a single layer, which is also the amount of substance adsorbed on the surface when the coverage is 1; and *c_e_* is the concentration of adsorbate in the solution at equilibrium. There is a direct relationship between the adsorption equilibrium constant *K* and the adsorption energy.

(2) ΔG=−RTlnK

(3) $$K=exp(-\frac{\Delta G}{RT})$$

It can be observed that surface coverage is proportional to solution concentration *c*_e_ only when the solution concentration is extremely low, often in the ppm range [11], or when the surface adsorption energy is extremely low. However, in terms of corrosion, the adsorption capacity of adsorbates is often strong, and the concentration is much higher than the ppm order of magnitude.

Currently, in the construction process of conventional first-principles computational models, due to the influence of model size, the coverage of adsorbates often adopts characteristic values such as 1, 1/2, 1/4, etc., and it is impossible to construct corresponding coverage based on the actual concentration of the solution system. This limits the flexibility in simulating surface properties under different concentration conditions. To address this, the traditional formulas such as the Nernst equation are applied to simulate the effect of concentration changes on surface properties, while keeping the basic first-principles computational model unchanged.

It is worth noting that in solution, adsorbates of different concentrations can significantly alter the charge shielding effect. Even without modifying the model structure itself, this change may further affect the surface properties of materials. Previous studies have often overlooked this point. This study will focus on two typical metal materials to explore how their surface properties change under different adsorption conditions to fill the gaps in existing research and provide a deeper understanding.

## 2. Results

### 2.1. Surface Adsorption Configuration

**Figure 1:**
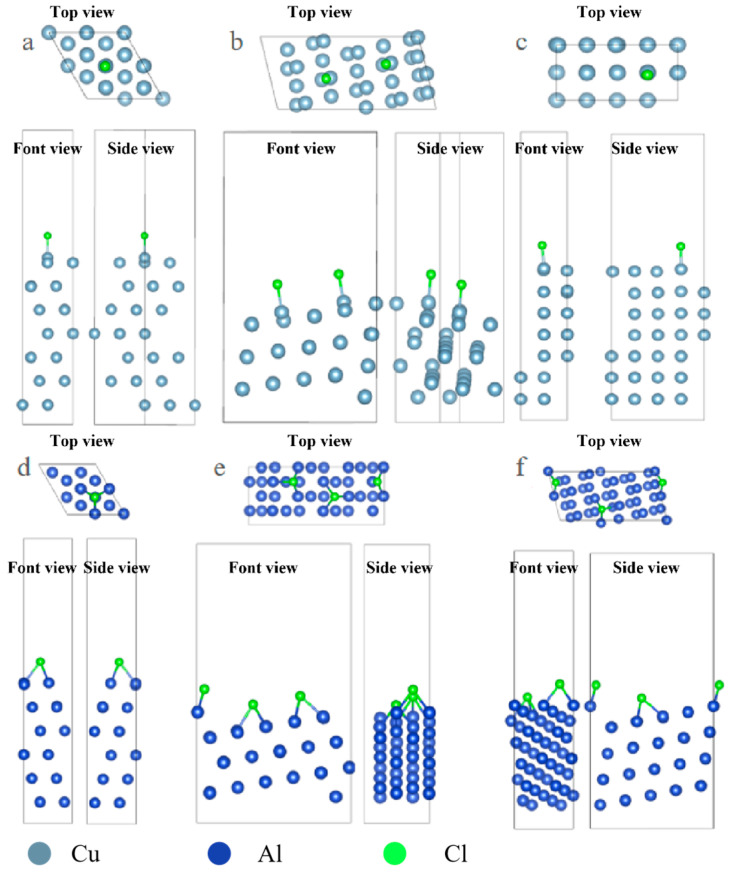
Three-view diagrams of the configurations of three metals after the adsorption of Cl on different surfaces. (**a**) Al(111); (**b**) Al(322); (**c**) Al(100); (**d**) Cu(111); (**e**) Cu(322); (**f**) Cu(332).

Under the condition of consistent surface coverage, the adsorption configurations of surface Cl ions and S ions on the surface were optimized, and the results are shown in Figure 1.

**Figure 2:**
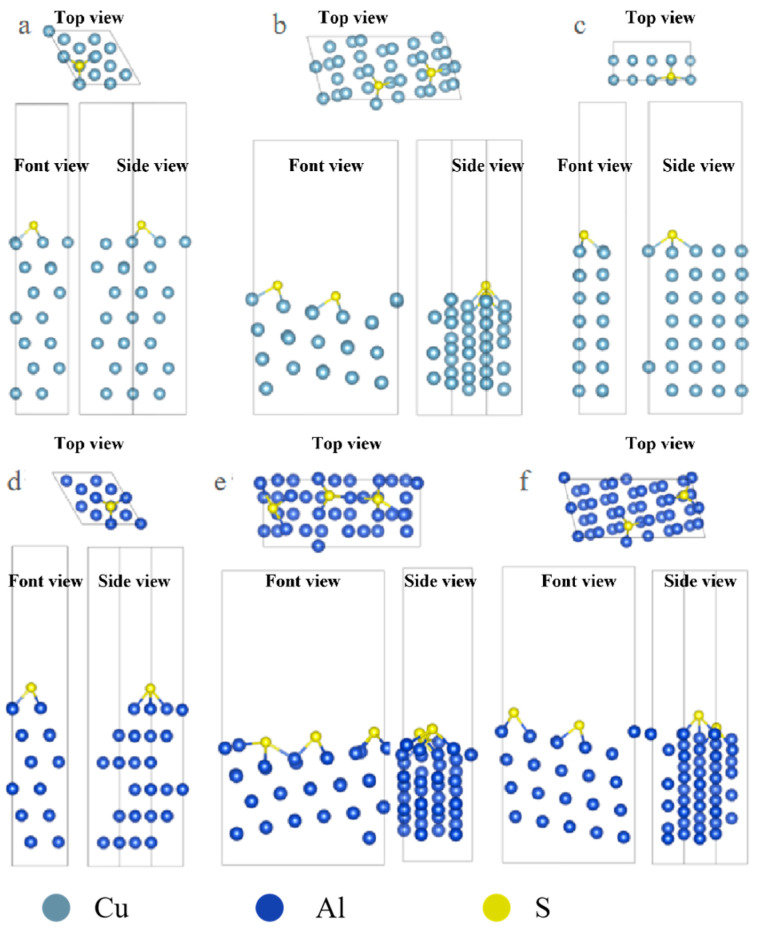
Three-view diagrams of the configurations of three metals after the adsorption of S on different surfaces. (**a**) Al(111); (**b**) Al(322); (**c**) Al(100); (**d**) Cu(111); (**e**) Cu(322); (**f**) Cu(332).

The optimized configuration of S atom adsorption is shown in Figure 2. From the image, it can be observed that the adsorption configuration of S on some surfaces is significantly different from that of Cl and S with more empty orbitals, and it tends to combine with more metal atoms. In the case of Cl adsorption on the Al surface, it mostly adsorbs onto the Top sites, while S adsorbs more onto the Hollow sites.

Subsequently, the average adsorption energy and work function of its surface can be further calculated, with the results presented in Table 1 and Table 2. The formula for calculating adsorption energy is as follows:

(4) $$E_{\mathrm{adsorption}}=\frac{E_{\mathrm{adsorbate}+\mathrm{surface}}-(E_{\mathrm{surface}}+nE_{\mathrm{adsorbate}})}{n}$$

where *E*_adsorption_ is the average adsorption energy per adsorbate, *E*_adsorbate+surface_ is the total energy of the entire adsorption system, *E*_surface_ is the energy of the pure surface without any adsorbed atoms, and *E*_adsorbate_ is the energy of the individual adsorbate.

This formula calculates the difference between the energy of the entire adsorption system and the energy of a clean surface plus the corresponding number of adsorbates.

Then, by dividing this difference by the number of adsorbates, the average contribution of each adsorbate to the total adsorption energy is obtained. It is evident that the calculated overall adsorption energy values are very low, indicating that atoms are easily adsorbed onto metal surfaces. Because gaseous single atoms are used as adsorbates in the calculation, this leads to a strong interaction between the metal surface and the atoms. In reality, adsorption often occurs in solutions or with adsorbates that have already formed molecules. However, this calculation can still reflect, to some extent, the relationship with corresponding pollutants. The lower the adsorption energy, the closer the interaction with the adsorbate. The variation pattern of adsorption energy reveals that the interaction with different pollutants follows the order of Al < Cu.

In addition, it is noteworthy that when adsorption occurs on stepped interfaces such as 322 and 332 surfaces, the distance between the adsorbed atoms and the steps is shorter than the average bond length. This indicates that the binding between the steps and the adsorbed atoms is more tight than on a flat surface. This result also suggests that stepped interfaces tend to be more prone to corrosion.

### 2.2. Concentration Difference Under Solvation

**Figure 3:**
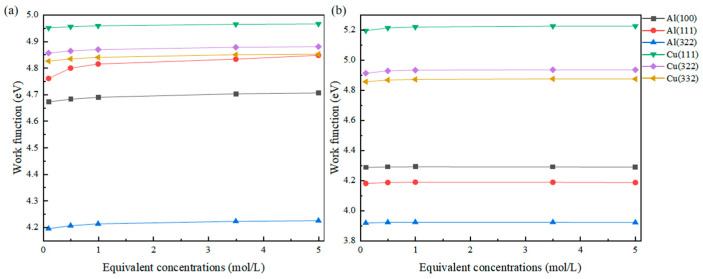
Changes in the work function of Cl (**a**) and S (**b**) adsorbed on different surfaces at different equivalent concentrations.

As mentioned earlier, in solutions of different concentrations, not only does the activity or coverage of adsorbed atoms change, but the overall dielectric properties of the solution are also altered. Therefore, by incorporating an implicit solvent model and setting different Debye lengths to simulate the changes in the dielectric properties of the solution, the results obtained based on this are shown in Figure 3.

It can be observed that as the concentration increases, the change in dielectric properties of the solution leads to a significant change in work function. For the most obvious example, the work function of the Al(111) surface adsorbed with Cl at an equivalent concentration of 0.1 mol/L is 4.761 eV, while at an equivalent concentration of 5 mol/L, the work function reaches 4.848 eV, showing a difference of nearly 90 meV. The work function differences for the other surfaces are also around 30 meV.

**Figure 4:**
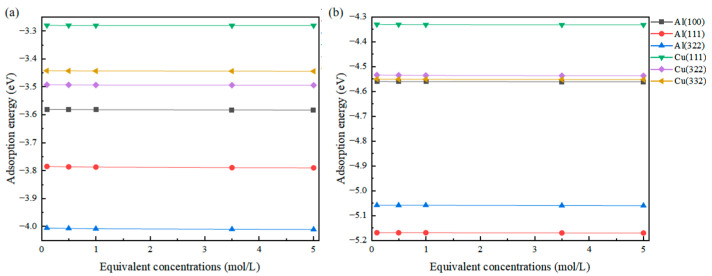
Changes in the adsorption energy of Cl (**a**) and S (**b**) adsorbed on different surfaces at different equivalent concentrations.

The results obtained are presented in Figure 4, showing that compared to the work function, the change in adsorption energy is not as pronounced. This is because the adsorption energy primarily originates from the direct strong interaction between the adsorbed atom and the surface atom. There exists a high-concentration charge region between them, and the external charge shielding effect struggles to affect the bonding between atoms, making the change in equivalent concentration have little impact on the adsorption energy. Unlike this, the relationship between work function and equivalent concentration is different. The definition of work function and the “vacuum energy level” in a solution environment are significantly affected by changes in the dielectric properties of the solution.

Comparative analysis of work functions between non-solvated and solvated models at 1 mol/L reveals distinct trends (Table 3 and Table 4). The most significant work function variation occurs for Cl atoms adsorbed at the Top site on Al surfaces, showing the largest adsorption-induced shift. Substituting Cl with S alters the preferred adsorption sites on Al surfaces from Top to Hollow and Bridge configurations, accompanied by substantial solvent-induced work function modifications. This structural reorganization results in more pronounced adsorption geometry changes with reduced adsorbate-surface distances, consequently enhancing work function shifts. Other adsorption systems display minimal solvent effects, with work function variations remaining below 0.1 eV upon solvation.

## 3. Models and Computational Methods

### 3.1. Computational Methods

In this study, Vienna Ab initio Simulation Package (VASP/5.4.4) [12] was used for first-principles calculations, simulating the electronic structure of periodic solids through the plane wave basis set and the Projector Augmented Wave (PAW) method. In this computation, the Perdew–Burke–Ernzerhof (PBE) functional [13] was used to describe the exchange-correlation energy. To ensure the accuracy of the computational results, the energy cutoff of the wavefunction was tested and ultimately an appropriate cutoff energy of 500 eV was selected. Regarding k-point sampling, the kspacing parameter was used to determine the density of the k-point grid by specifying the distance between k-points. The kspacing was set to 0.25 Å^−1^, ensuring dense sampling of the Brillouin zone and thereby improving the accuracy of the computational results. The occupation of electronic states was smoothed using Fermi–Dirac distribution. The convergence criterion for self-consistent field (SCF) calculations was set to 10^−5^ eV to ensure high-precision convergence of electronic energy. Structural optimization was performed through force convergence calculations, employing a force convergence criterion of 0.01 eV/Å.

To more accurately simulate the behavior of the system in a solution environment, the VASPsol/1.0 implicit solvent model [14,15] was used. VASPsol is an implicit solvent model based on continuum theory, which simulates the influence of the solution environment on the system by incorporating a description of the solvent medium in the calculation. In this study, default aqueous medium parameters were used with a dielectric constant set to 78.4 to simulate the aqueous solution environment. By incorporating the VASPsol model in the VASP calculations, it was able to more accurately describe the influence of the solution environment on the electronic structure and energy, thereby obtaining more reliable computational results.

### 3.2. Computational Parameters

In the parameter settings of the implicit solvent model in VASPsol, the parameters and their corresponding physical meanings are shown in Table 5. For the relative dielectric constant EB_K of solvent molecules, since almost all solvent molecules in actual corrosion environments are water molecules, this parameter will use the default value and will not be changed.

The settings for variables SIGMA_K and NC_K are parameters related to dielectric holes. In the relevant paper [14], the developers propose the following hypothesis: a diffusing dielectric hole is a local function of the solute’s charge density, that is *ε*(r), a function of the relative dielectric constant $n_{\mathrm{solute}(r)}$. This hypothesis leads to a diffusion hole implicitly determined by the electronic structure of the solute. The smooth transition to the hole also ensures that the derivative of the energy functional is continuous, simplifying the implementation of geometric optimization for the solute system. The functional dependence of the solvent’s relative dielectric constant on the solute’s electronic charge density is assumed to follow:

(5) $$\varepsilon (n_{\mathrm{solute}(r)})=1+(\varepsilon_{b}-1)S(n_{\mathrm{solute}(r)})$$

where $\varepsilon_{b}$ is the relative dielectric constant of the bulk solvent and $n_{\mathrm{solute}(r)}$ is the pore shape function. The definition of the hole shape function is as follows:

(6) $$S\left(n_{\mathrm{solute}}(r)\right){=}^{1}{/}_{2}\mathrm{erfc}\left(\frac{log(n_{\mathrm{solute}}/n_{c})}{\sigma \sqrt{2}}\right)$$

where $n_{c}$ determines how much charge density can form dielectric holes, while σ determines the width of the diffusion holes. Specifically, an increase in the value will increase the width of the cavity, making the transition from electron density to dielectric constant change smoother.

The variables NC_K and SIGMA_K correspond to the aforementioned parameters, respectively. As NC_K and SIGMA_K increase, the formed dielectric holes will become larger, the transition region will become wider, and the solvated holes will become more stable. When modifying the solvation model, it is numerically more stable to change SIGMA_K while keeping the variable NC_K at its default value. According to the tests conducted by Wang et al. [16], as NC_K and SIGMA_K increase, the solvation energy becomes more negative, and the intermediate becomes more stable due to the solvation effect. However, too-large NC_K or SIGMA_K can lead to unrealistic stability of the intermediate, which is consistent with previous understanding of the formula. Therefore, the two parameters remain unchanged to avoid generating computationally unstable results.

During the parameter-setting process, not all parameters are optimized by choosing the default values. According to the research conducted by Gauthier et al. [17], when calculating the solvated surface of Pt(111), setting TAU to the default value of 0.000525 V Å^−2^ results in noticeable fluctuations at the electrode–solution interface, and there are also minor sawtooth-like fluctuations away from the interface. This can make it difficult to determine the electrostatic potential of the bulk solution during the calculation and increases the instability of the calculation. However, when TAU is set to 0, the obtained electrostatic potential distribution shows no significant error in overall numerical values compared to the default value, but it significantly eliminates the overall numerical instability and fluctuation. Therefore, in future calculations, the setting value of TAU will be determined as 0.

The parameter LAMBDA_D_K represents the Debye length of ions in a solution, which is related to concentration and valence. The Debye length can indicate the typical range of charge fluctuation shielding in a medium, which is a fundamental concept in electrolyte theory. In an ideal situation, charge fluctuation decreases to 1/*e*, approximately 37% of its original value at a distance of *λ_D_*. Therefore, the Debye length determines the interaction distance of charges in a medium. The specific formula is as follows:

(7) $$\lambda_{D}={\left(\frac{\varepsilon k_{B}T}{\sum_{j=1}^{N}n_{j}^{0}q_{j}^{2}}\right)}^{1/2}$$

where *ε* is the dielectric constant, *ε* = *ε*_0_*ε*_r_; *ε*_0_ = 8.85 × 10^−12^ F/m is the permittivity of the vacuum; *ε*_r_ = 80.1 is the relative permittivity of water at room temperature; *k_B_* is the Boltzmann constant; *T* is the temperature; $n_{j}^{0}$ is the concentration of the *i*th ion; *q_j_* is the charge of the *i*th ion; and *N* is the total number of different types of ions in the system, and by modifying this parameter, solution environments with different concentrations can be simulated. In addition, there are also some additional parameters for calculation, such as the charge of an electron *e* = 1.6 × 10^−19^ C and the Avogadro constant *N_A_* = 6.022 × 10^23^. Then, for different concentrations, we can further calculate the corresponding Debye length in the solution environment according to the formula.

For the conventional double-ion solution environment, the formula is simplified to

(8) $$\lambda_{D}={\left(\frac{\varepsilon_{0}\varepsilon_{r}k_{B}T}{{z_{a}}^{2}e^{2}c_{a}N_{A}+{z_{c}}^{2}e^{2}c_{c}N_{A}}\right)}^{1/2}$$

where *z_c_*, *c_c_*, *z_a_*_,_ and *c*_a_ represent the charge of cations, concentration of cations, charge of anions, and concentration of anions, respectively. Further calculations are performed to obtain the corresponding Debye length and modify the influence of the solution environment on surface adsorption. The Debye lengths calculated for common erosive ions Cl and S atoms at different concentrations in this study are shown in Table 6.

### 3.3. Modeling

**Figure 5:**
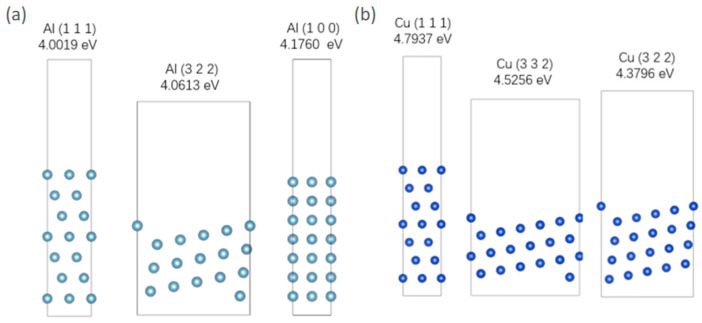
(**a**) Three lowest energy surface models of Al; (**b**) three lowest energy surface models of Cu.

The construction of this model involves searching for different orientations and surface energy densities of Al and Cu in the Materials Project. The results are presented in Table 7. Surface energy represents the energy required to break intermolecular forces when creating a material surface. Therefore, the higher the surface energy density, the lower the probability and proportion of the surface appearing. Therefore, selecting the three crystal-plane orientations with the lowest surface energy can represent the surface conditions of metals to the greatest extent. A vacuum layer of 15 Å is added to the surface when constructing the model. The optimized model structure is shown in Figure 5. It is important to acknowledge a key simplification in the adsorption mode. In realistic aqueous corrosion environments, sulfur is typically present in anionic forms such as HS^−^, S^2−^, or SO_4_^2−^. Atomic S is used in this study instead of anionic S. This choice is made for computational tractability and consistency across comparative simulations. Therefore, the results presented here should be interpreted in terms of relative trends.

After constructing the surface models, the coverage of the surface is set at one-quarter to study the influence of concentration on surface adsorption from the perspective of solution dielectric properties. Based on the number of surface adsorption sites, we can further determine the number of molecules adsorbed on the surface. Specifically, one Cl/S is adsorbed on the Al(111) and Al(100) surfaces, two Cl/S are adsorbed on the Al(322) surface, one Cl/S is adsorbed on the Cu(111) surface, three Cl/S are adsorbed on the Cu(332) surface, and two Cl/S are adsorbed on the Cu(322) surface.

## 4. Conclusions

(1) The concentration of solute in the solvent not only affects the coverage of adsorbate on the surface but also affects the Debye length of the solvent environment on the surface, thereby altering the work function of the surface.
(2) In the presence of added solvation, the adsorption energy between the adsorbed atom and the surface does not undergo significant changes due to the effect of strong bonding, whereas the work function monotonically increases with the increase in equivalent concentration. The work function of the Al(111) surface shows an increase of approximately 90 meV as the equivalent concentration of adsorbed Cl increases from 0.1 mol/L to 5 mol/L.
(3) The solvation environment is highly sensitive to the adsorption configuration. The adsorbed molecule at the Top site is the furthest from the surface and is most significantly influenced by electrostatic shielding in the solution, resulting in the largest amplitude of work function change.

## Figures and Tables

**Table 1:** Surface properties of different metals when adsorbing Cl at the minimum energy.

Metal and Surface Orientation	Surface Work Function (eV) Without Adsorption	Adsorption Sites	Average Bond Length (Å) After Adsorption	Surface Work Function (eV) After Adsorption	Difference in Work Function (eV) Before and After Adsorption	Adsorption Energy (eV)
Al(111)	4.001	Top	2.179	5.686	1.684	−3.643
Al(322)	4.061	Top	2.173	5.428	1.366	−3.871
Al(100)	4.176	Top	2.169	5.316	1.140	−3.506
Cu(111)	4.711	Hollow	2.396	5.319	0.607	−3.277
Cu(332)	4.525	Hollow	2.378	5.457	0.931	−3.426
Cu(322)	4.379	Hollow	2.367	5.470	1.090	−3.479

**Table 2:** Surface properties of different metals when adsorbing S at the minimum energy.

Metal and Surface Orientation	Surface Work Function (eV) Without Adsorption	Adsorption Sites	Average Bond Length (Å) After Adsorption	Surface Work Function (eV) After Adsorption	Difference in Work Function (eV) Before and After Adsorption	Adsorption Energy (eV)
Al(111)	4.001	Hollow	2.349	4.482	0.480	−5.277
Al(322)	4.061	Hollow	2.353	4.360	0.298	−5.188
Al(100)	4.176	Bridge	2.320	4.544	0.368	−4.788
Cu(111)	4.711	Hollow	2.213	5.508	0.797	−4.497
Cu(332)	4.525	Hollow	2.221	5.341	0.815	−4.846
Cu(322)	4.379	Hollow	2.214	5.441	1.061	−4.647

**Table 3:** Changes in work function before and after solvation for Cl atom adsorption on the lowest energy surface of different metals.

Metal and Surface Orientation	Surface Work Function (eV) Without Adsorption	Adsorption Sites	Average Bond Length (Å) After Adsorption	Surface Work Function (eV) After Adsorption	Solventization Work Function (eV)	Work Function Difference (eV)
Al(111)	4.001	Top	2.179	5.686	4.815	−0.870
Al(322)	4.061	Top	2.173	5.428	4.214	−1.213
Al(100)	4.176	Top	2.169	5.316	4.690	−0.625
Cu(111)	4.711	Hollow	2.396	5.319	4.958	−0.360
Cu(332)	4.525	Hollow	2.378	5.457	4.840	−0.616
Cu(322)	4.379	Hollow	2.367	5.470	4.869	−0.600

**Table 4:** Changes in work function before and after solvation for S atom adsorption on the lowest energy surface of different metals.

Metal and Surface Orientation	Surface Work Function (eV) Without Adsorption	Adsorption Sites	Average Bond Length (Å) After Adsorption	Surface Work Function (eV) After Adsorption	Solventization Work Function (eV)	Work Function Difference (eV)
Al(111)	4.001	Hollow	2.349	4.482	4.190	−0.292
Al(322)	4.061	Hollow	2.353	4.360	3.925	−0.435
Al(100)	4.176	Bridge	2.320	4.544	4.292	−0.251
Cu(111)	4.711	Hollow	2.213	5.508	5.219	−0.289
Cu(332)	4.525	Hollow	2.221	5.341	4.871	−0.469
Cu(322)	4.379	Hollow	2.214	5.441	4.933	−0.507

**Table 5:** Default parameters for implicit solvent models and corresponding interpretations.

Parameters	Default Value	Interpretation
LSOL	TRUE	Enable solventization model functionality
EB_K	78.4	The relative dielectric constant used to describe solvent molecules, defaulting to water
SIGMA_K	0.600	Width of dielectric holes
NC_K	0.0025	Charge density cutoff value at dielectric holes
TAU	0.000525	Surface tension related to non-electrostatic forces at dielectric holes
LAMBDA_D_K	3.0	In Å units, used to describe the Debye length of ions in solution, which is related to concentration and valence

**Table 6:** Correlation between concentration and Debye length in two typical solutions.

Solutions	Concentration (mol/L)	Debye Length (Å)
NaCl	0.1	9.71
	0.5	4.35
	1	3.07
	3.5	1.64
	5	1.37
Na_2_S	0.1	4.35
	0.5	1.94
	1	1.37
	3.5	0.73
	5	0.61

**Table 7:** Surface energy of two typical metals with different cross-sections.

Al		Cu	
(hkl)	Surface Energy (J·m^−2^)	(hkl)	Surface Energy (J·m^−2^)
(111)	0.769	(111)	1.339
(322)	0.903	(332)	1.435
(100)	0.906	(322)	1.454
(332)	0.909	(100)	1.47
(221)	0.948	(221)	1.478
(331)	0.96	(331)	1.516
(321)	0.964	(311)	1.54
(311)	0.976	(110)	1.561
(110)	0.977	(321)	1.578
(211)	0.979	(310)	1.589
(310)	0.994	(210)	1.597
(320)	1.013	(320)	1.621
(210)	1.015	(211)	1.626

## Data Availability

Data will be made available on request.
